# Correlation between skeletal muscle acetylcarnitine and phosphocreatine metabolism during submaximal exercise and recovery: interleaved ^1^H/^31^P MRS 7 T study

**DOI:** 10.1038/s41598-024-53221-x

**Published:** 2024-02-08

**Authors:** Radka Klepochová, Fabian Niess, Martin Meyerspeer, Dorota Slukova, Ivica Just, Siegfried Trattnig, Jozef Ukropec, Barbara Ukropcová, Alexandra Kautzky-Willer, Michael Leutner, Martin Krššák

**Affiliations:** 1 Division of Endocrinology and Metabolism, Department of Internal Medicine III, Medical University of Vienna, Währinger Gürtel 18-20, 1090 Vienna, Austria; 2 High-Field MR Center, Department of Biomedical Imaging and Image-Guided Therapy, Medical University of Vienna, Vienna, Austria; 3 High-Field MR Center, Center for Medical Physics and Biomedical Engineering, Medical University of Vienna, Vienna, Austria; 4 Christian Doppler Laboratory for Clinical Molecular MR Imaging (MOLIMA), Vienna, Austria; 5 Institute of Experimental Endocrinology, Biomedical Research Center, Slovak Academy of Sciences, Bratislava, Slovakia

**Keywords:** Biological physics, Medical research, Biomarkers

## Abstract

Acetylcarnitine is an essential metabolite for maintaining metabolic flexibility and glucose homeostasis. The in vivo behavior of muscle acetylcarnitine content during exercise has not been shown with magnetic resonance spectroscopy. Therefore, this study aimed to explore the behavior of skeletal muscle acetylcarnitine during rest, plantar flexion exercise, and recovery in the human gastrocnemius muscle under aerobic conditions. Ten lean volunteers and nine overweight volunteers participated in the study. A 7 T whole-body MR system with a double-tuned surface coil was used to acquire spectra from the gastrocnemius medialis. An MR-compatible ergometer was used for the plantar flexion exercise**.** Semi-LASER-localized ^1^H MR spectra and slab-localized ^31^P MR spectra were acquired simultaneously in one interleaved exercise/recovery session. The time-resolved interleaved ^1^H/^31^P MRS acquisition yielded excellent data quality. A between-group difference in acetylcarnitine metabolism over time was detected. Significantly slower τ_PCr recovery_, τ_PCr on-kinetics_, and lower Q_max_ in the overweight group, compared to the lean group was found. Linear relations between τ_PCr on-kinetics_, τ_PCr recovery_, VO_2max_ and acetylcarnitine content were identified. In conclusion, we are the first to show in vivo changes of skeletal muscle acetylcarnitine during acute exercise and immediate exercise recovery with a submaximal aerobic workload using interleaved ^1^H/^31^P MRS at 7 T.

## Introduction

Acetylcarnitine can be detected in skeletal muscle tissue at 2.13 ppm by proton magnetic resonance spectroscopy (^1^H MRS)^1,2^ and has recently been suggested to be a very important metabolite in the control of muscle oxidative metabolism, glucose homeostasis, and insulin sensitivity^3–6^. The availability of free carnitine is recognized as a crucial factor in acetylcarnitine formation and the maintenance of metabolic flexibility^3,4^. The excessive buildup of acetyl-CoA serves as a strong allosteric inhibitor of pyruvate dehydrogenase, which serves as a key enzyme controlling the entry of pyruvate into the Krebs cycle^7^. The interconversion of acetyl-CoA and acetylcarnitine is catalyzed via the mitochondrial matrix enzyme carnitine acetyltransferase (CrAT)^8^. Although invasive measurements of acetylcarnitine following intensive exercise are well established^9–14^, there are only few reports on non-invasive dynamic measurements^2,3^.

Differences in baseline (non-exercise) acetylcarnitine concentrations have been shown between triathletes, active healthy individuals with normal glucose tolerance, patients with impaired glucose tolerance, and patients with type 2 diabetes mellitus (T2DM)^15,16^. It has been shown that a heavy workout (high-intensity exercise) leads to an immediate post-exercise increase of acetylcarnitine concentrations^17^. However, the in vivo dynamics of muscle acetylcarnitine content during exercise and early recovery in populations with different levels of cardiometabolic fitness has not yet been examined.

^31^P MRS is a well‐established method with which to investigate skeletal muscle energy metabolism in vivo^18^. Physiologically relevant parameters can be derived from time courses of phosphocreatine (PCr) and inorganic phosphate (Pi), that is, pH, the time constant of PCr resynthesis (τ_PCr recovery_), the time constant of PCr decay at the onset of exercise (τ_PCr on-kinetics_), and maximum oxidative capacity, Q_max_^19–21^, which are linked to energy efficiency during contraction (exercise period). In particular, the τ_PCr recovery_ dynamics and Q_max_ are good measures of intracellular oxidative ATP production^22^. PCr-on-kinetics is assumed to be a marker of skeletal muscle mitochondrial inertia^21^. A prolonged duration signifies an extended dependence on PCr for ATP production, leading to a heightened skeletal mitochondrial inertia. The rate at which PCr is replenished serves as an indicator of oxidative capacity. A quicker recovery of PCr post-exercise signifies swifter replenishment and, consequently, superior mitochondrial function in restoring cellular energy. Moreover, from static fully relaxed muscle ^31^P MRS, we can calculate concentrations of phosphomonoesters (PMEs), phosphodiesters (PDEs), and nicotinamide adenine dinucleotide phosphate (NADP).

Although the detection of acetylcarnitine using ^1^H single-voxel MRS and the exercise-challenged time course of PCr and Pi concentrations using dynamic ^31^P MRS are well established and there is a good consensus about their standardization^23,24^, the concurrent interleaved measurement^25^ of these metabolites has not yet been performed. Reproducing the exact same exercise load under identical metabolic conditions in two sequentially repeated measurements is notoriously difficult. Hence, an assessment of acetylcarnitine, PCr, and Pi time courses during a single exercise-recovery experiment increases the volunteer’s compliance with the experimental procedures and could improve potential correlation between the metabolites. Recent improvements in interleaved MRS methodology^25–29^ and an advanced double-tuned multi-channel surface RF coil^30^ are the best pre-requisites for such an experiment.

Based on our previous experiments we hypothesize qualitatively similar trends between acetylcarnitine and PCr during the rest/exercise/recovery period, and therefore we expect correlations between dynamics of those two processes. Moreover, based on findings published previously^2,15,31,32^, we hypothesize lower acetylcarnitine concentrations at rest in overweight and sedentary volunteers.

Thus, we aimed to explore the time-dependent behavior of acetylcarnitine in more detail during rest, plantar flexion exercise, and recovery in the human gastrocnemius muscle (GM) and PCr and Pi evolution under aerobic conditions during a single experiment using non-invasive interleaved ^1^H/^31^P MRS at 7 T. To demonstrate the physiologic value of this measurement, we have included two metabolically different groups of healthy volunteers (lean and active and overweight and sedentary). Last but not least, we aimed to perform a bivariate analysis of the interrelations between acetylcarnitine and ^31^P-derived parameters, as post-exercise phosphocreatine recovery rates had previously been shown to be positively associated with CrAT activity and coincided with dramatic shifts in muscle acetylcarnitine dynamics^33^.

## Methods

### Participant characteristics

Ten healthy, lean, and active volunteers (Age: 30 ± 6 years, BMI: 21.3 ± 2.4 kg/m^2^, sex: 7 f/3 m) and nine overweight and sedentary volunteers (Age: 33 ± 6 years, BMI: 32.7 ± 3.0 kg/m^2^, sex: 5 f/4 m) participated in the study. Forty-five to ninety minutes of continuous exercise, regardless of endurance or resistance training, was considered as one training unit. Written, informed consent was provided in accordance with the local ethics committee requirements. The study was approved by the ethics committee of the Medical University of Vienna (NR:1081/2020) and all experiments were performed in accordance to the guidelines of the Declaration of Helsinki.

To determine maximal oxygen uptake (VO_2max_), all participants underwent a standardized protocol with continuous increments, until exhaustion, on a cycle ergometer (Lode Excalibur, Groningen, The Netherlands). Measurement of VO_2max_ and other parameters was performed via "breath-by-breath" spiroergometry (Master CPX, VIASYS Healthcare).

The maximal voluntary contraction force (MVC) of the right leg was measured with a leg-press dynamometer (FPES CU, Bratislava, Slovakia) and served later to adjust the exercise load for the in-magnet challenge.

### Magnetic resonance (MR) measurements

**Figure 1:**
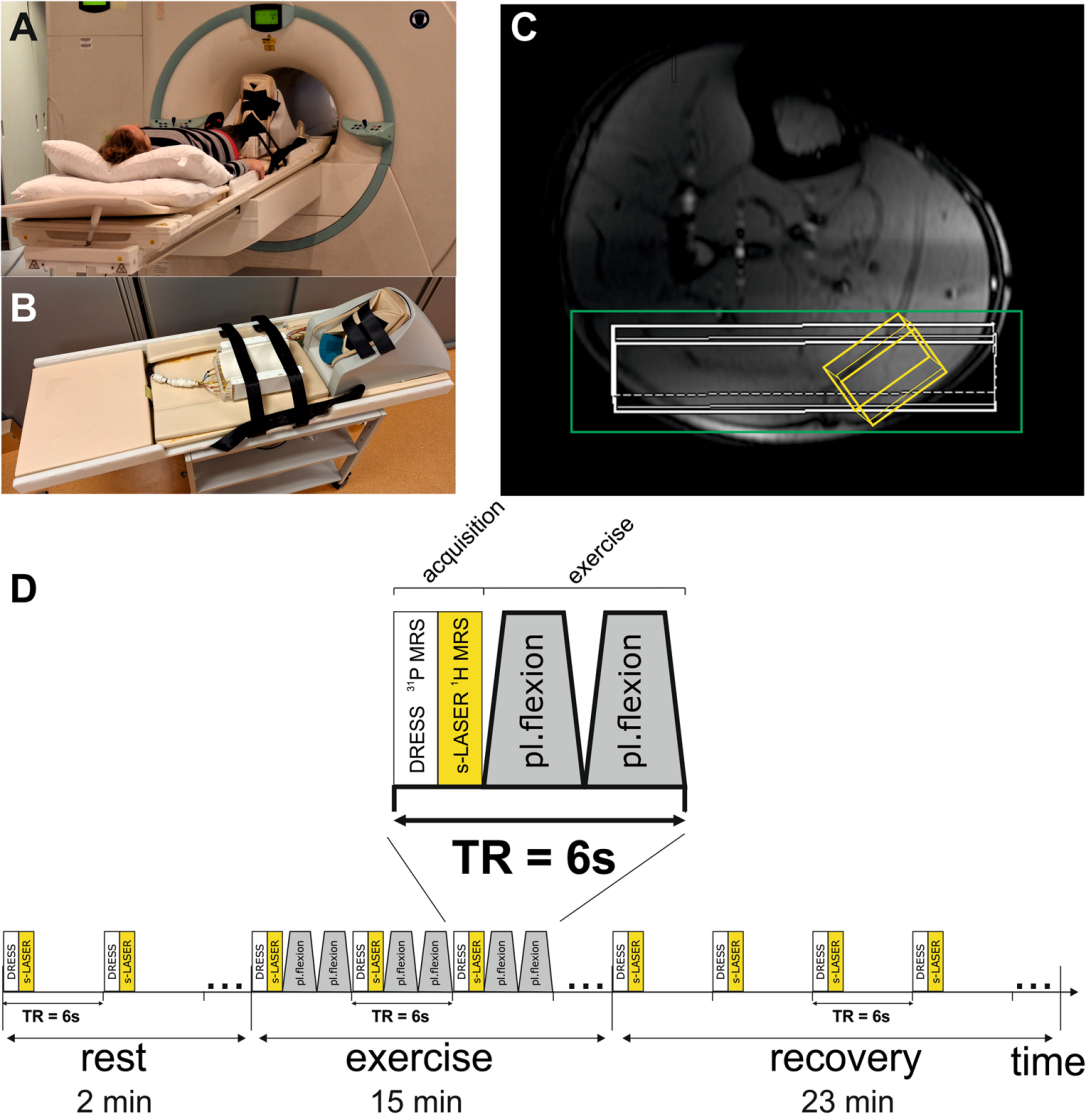
Whole setup of volunteer lying on the 7 T MR system table with right calf muscle placed on the double-tuned ^1^H/^31^P coil and on the ergometer pedal fixed with belts (**A**, **B**), Axial localizer image of a calf muscle with depicted slice selection of the DRESS localization sequence from ^31^P MRS (white slab), VOI for acetylcarnitine acquisition with ^1^H MRS (yellow voxel), and shimming volume (green rectangle) (**C**), interleaved rest/exercise/recovery session (2 min rest, 15 min exercise at 30–40% of MVC, 23 min recovery) and acquisition of DRESS-slab-localized ^31^P MR spectra from white slab and semi-LASER-localized ^1^H MR spectra from yellow voxel followed by plantar flexion exercise—twice pressed pedal within a TR of 6 s (**D**).

All MR measurements were performed on a 7 T whole-body MR system (Terra Dot Plus, Siemens Healthineers, Erlangen, Germany). A double-tuned surface coil transceiver array with two ^1^H channels (*d* = 17 cm, *l* = 12.5 cm) and three ^31^P channels (*d* = 15 cm, *l* = 10 cm shaped to the anatomy of the human calf^30^ was used to acquire spectra from the gastrocnemius medialis/lateralis of the right leg. An MR-compatible ergometer (Trispect, Ergospect, Innsbruck, Austria) was used for the plantar flexion exercise. The setup of coil and ergometer inside of the scanner is depicted in Fig. 1A and B.

Measurements were performed in the morning, after an overnight fast in a single exercise-recovery session. Volunteers were in the supine position with the right calf muscle placed on the double-tuned ^1^H/^31^P coil, on the ergometer, inside the 7 T MR system.

For volume-of-interest (VOI) positioning, localizer MR images were acquired. The VOI for ^1^H MRS acetylcarnitine detection (13 × 20 × 40 mm^3^) and the slab for ^31^P MRS PCr and Pi detection (thickness of 18 mm) were carefully placed predominantly within the gastrocnemius medialis and lateralis muscles (Fig. 1C). The linewidth of the water signal after shimming was in the range of 35–45 Hz, in magnitude spectra.

First, fully relaxed, static long-TR ^31^P MR spectra (TR = 15 s, NA = 8) were obtained.

Afterwards, semi-LASER^34,35^ localized ^1^H MR spectra (TE = 300 ms) (acetylcarnitine) and slab-localized (DRESS) (^31^P MR spectra (PCr, Pi)^36^ were acquired in an interleaved fashion, in a single exercise/recovery session (2 min rest, 15 min exercise at 30–40% of MVC, 23 min recovery) with a TR of 6 s, similar to what has already been published^27^ (Fig. 1D). Volunteers were instructed to press the pedal twice within every TR, with the noise of the spoiler gradients serving as an audio cue, to ensure data acquisition in a relaxed state of the muscle. For absolute quantification of acetylcarnitine, the fully relaxed water signal was measured separately (TR = 6000 ms, TE = 30 ms).

### Post processing

All ^1^H/^31^P spectroscopy data were extracted and processed from raw data using in-house-developed Python scripts (http://www.python.org) for phasing and channel combination. Signals were phased to the highest peak magnitude of PCr, water, or lipids in the frequency domain after 7 Hz Lorentzian apodization and 2 × zero-filling. The channel combination was then performed by weighted averaging of the raw data (that is, without apodization and zero-filling).

All spectra were analyzed with the fitting routine AMARES, using jMRUI v6.0 alpha, and acetylcarnitine concentrations were calculated as described in^16^ in mmol/L tissue volume units (further mmol/L only). In all 19 volunteers, we evaluated a time series of 400 spectra. For the further post hoc tests, we averaged 20 blocks at each of the following time intervals: rest (0–1st minute); middle of exercise (7th–8th minute); end of exercise (15th–16th minute); recovery (18th–19th minute); 10 min of recovery (27th–28th minute); 15 min of recovery (32nd and 33rd minute); and end of recovery (38th–39th minute). Moreover, we calculated the difference between acetylcarnitine concentrations from the end of the recovery period to the middle of exercise (Δ[Ac] _recovery-exercise_). IMCL CH_2_ at 1.3 ppm was corrected for T_1_ and T_2_ relaxation effects and expressed as a percentage of water content.

The static, fully relaxed ^31^P MR spectra provided concentrations of PCr, Pi, NADP, PDEs-glycerolphoshocholine (GPC) and glycerolphosphoethanolamine (GPE), and PMEs-phosphocholine (PC) and phopshoethanolamine (PE) in mmol/L units. The interleaved/dynamic ^31^P MRS protocol yielded the rate of PCr depletion during the exercise (τ_PCr on-kinetics_), the rate of PCr resynthesis during recovery (τ_PCr recovery_), the maximal rate of oxidative phosphorylation, i.e., mitochondrial capacity (Q_max_), the initial recovery rate (V_PCr_), and the time course of intracellular pH changes in skeletal muscle^37,38^. The chemical shift between PCr and Pi was used to calculate intramyocellular pH. ADP levels were calculated assuming creatine kinase (CK) to be at equilibrium (with K_CK_ = 1.66 × 10^9^ M^–1^) and upon the assumptions that PCr represents 85% of the total creatine concentration at rest and [ATP] was equal to 8.2 mmol/L^39^. To calculate the time constant of τ_PCr on-kinetics_ and τ_PCr recovery_, [PCr] time courses were fitted with a mono-exponential function using curve fitting in MATLAB. V_PCr_ and Q_max_ were calculated as described in Valkovic et al.^24,38^.

### Statistical analysis

Data were tested for normality, the differences between groups, and the differences in the values of the acetylcarnitine concentration during rest-exercise-recovery. Changes were tested for significance by a general linear model repeated measures with tests of within-subjects effects (over time period during rest-exercise-recovery) and tests of between-subjects effects over time (between two groups). Additionally, Bonferroni correction per time point as post hoc tests were performed. All analyses were done in SPSS (version 28.0; IBM SPSS, Chicago, Illinois, USA). The relationships between metabolic parameters determined using ^1^H and ^31^P MRS were analyzed by linear correlations using Pearson’s correlation coefficient (two-tailed probability values) to estimate the strength of the relationship. The correlation coefficient of an absolute value of 0.46, which corresponded to a 95% confidence agreement, was taken as significant. All values are provided as mean ± standard deviations and a p value < 0.05 was considered significant.

### Ethics approval and consent to participate

Written, informed consent and MR safety forms signed by the participants was provided in accordance with the local ethics committee requirements (Ethikkommission der Medizinischen Universität Wien, EK Nr. 1081/2020).

## Results

### Participant characteristics

Participant characteristics are shown in Table 1. Overweight and sedentary volunteers had significantly higher BMI, lower VO_2max,_ and lower MVC.

**Table 1:** Participant characteristics.

	Lean and active (n = 10)		Overweight and sedentary (n = 9)		p-values
Age (years)	30 ± 6		33 ± 6		**< 0.001**
BMI (kg/m^2^)	**21.3 ± 2.4**		**32.7 ± 3.0**		**< 0.001**
VO_2max_ (mL/min/kg)	**39.55 ± 5.05**		**20.71 ± 4.87**		**< 0.001**
	Male (3)	Female (7)	Male (4)	Female (5)	
	45.23 ± 3.89	37.11 ± 3.16	24.73 ± 0.88	17.50 ± 4.23	
MVC (N)	**911 ± 142**		**518 ± 102**		

Significant values are in bold.

### MR measurement

**Figure 2:**
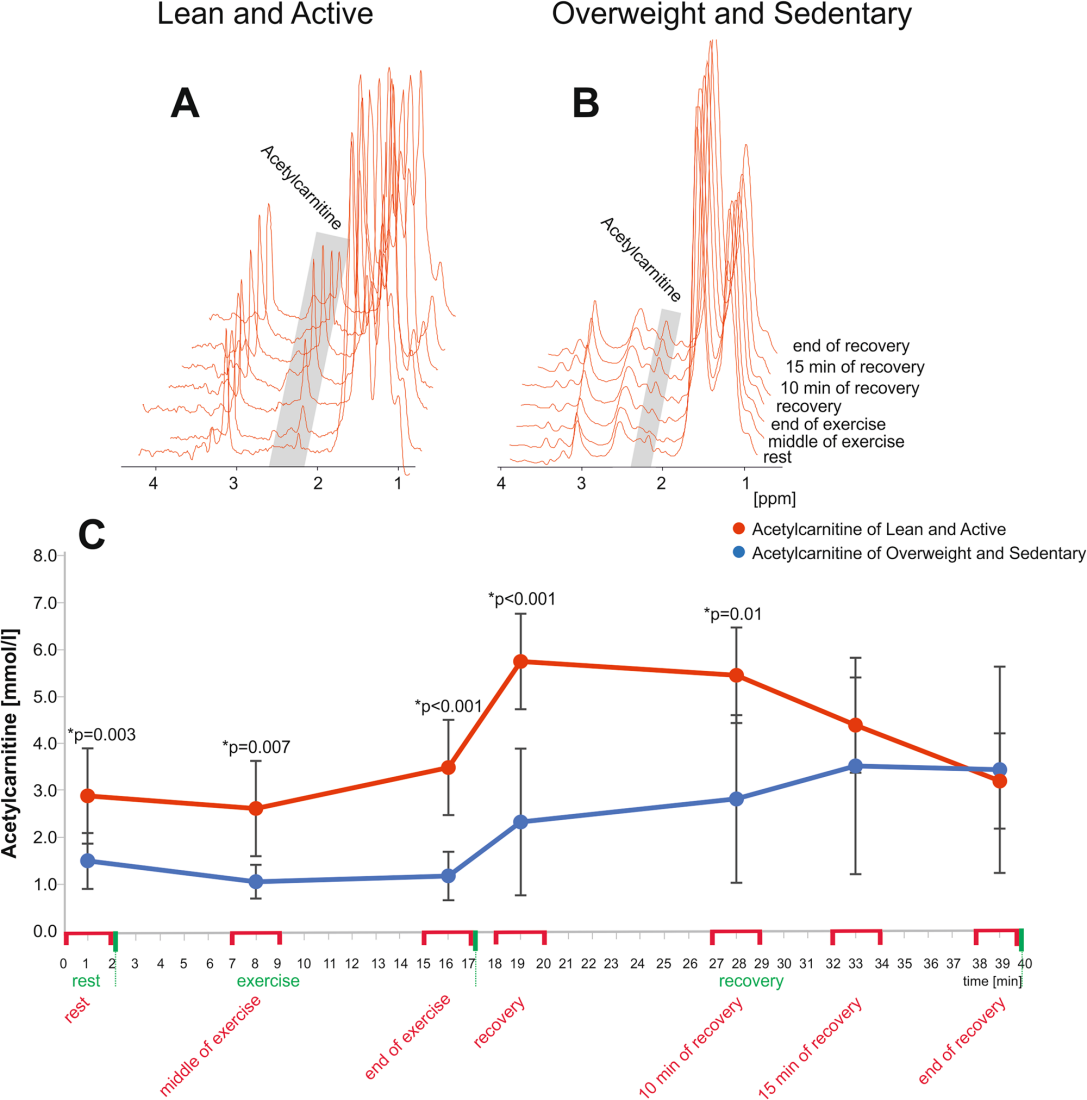
Representative average of 20 scans of ^1^H spectroscopy (acetylcarnitine acquisition) acquired in an interleaved ^1^H/^31^P MRS session during 2 min at rest, 15 min of plantar flexion exercise, and 23 min of recovery from GM of one volunteer from the lean and active (**A**) and one from the overweight and sedentary group (**B**). Representative time series of acetylcarnitine [mmol/L] evolution during exercise/recovery from two groups (**C**). On the timeline, the red colour marks exact minutes used to average the acetylcarnitine data.

**Figure 3:**
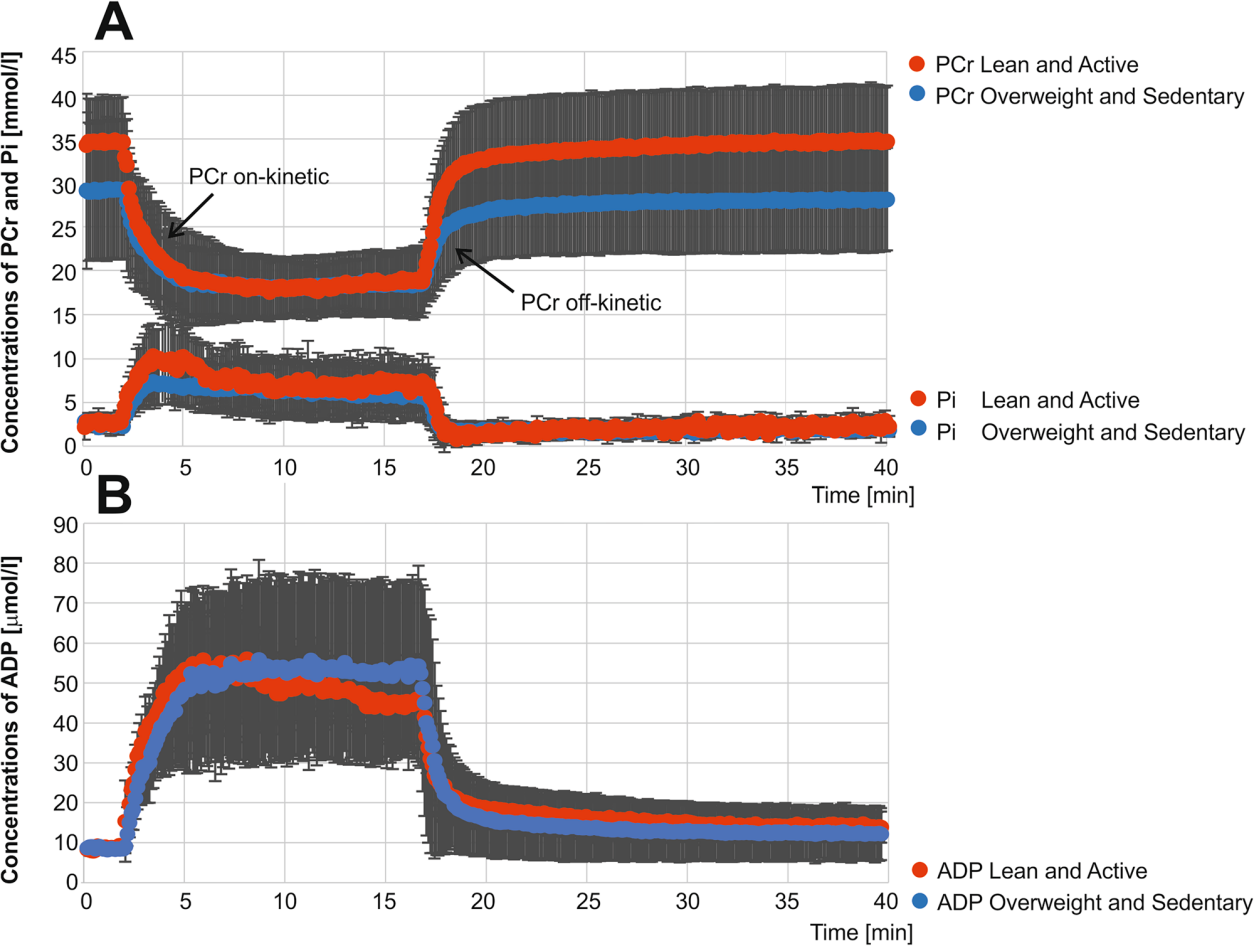
PCr [mmol/L], Pi [mmol/L] (**A**), and ADP [µmol/L] (**B**) evolution during the exercise/recovery challenge. Averages of all lean and active (red) and overweight and sedentary (blue) volunteers are shown.

All volunteers accepted the timing and exercise load prescribed in the protocol. The time-resolved interleaved ^1^H/^31^P MRS acquisition yielded excellent data quality (Figs. 2 and 3). Acetylcarnitine linewidth was 16.42 ± 4.21 Hz. Numerical results derived from both ^1^H and ^31^P data are summarized in Table 2.

**Table 2:** Results from ^1^H and ^31^P MRS.

	Lean and active (n = 10)	Overweight and sedentary (n = 9)	p-values
^1^***H*** ***MRS***			
[Ac] _at rest_ (mmol/L)	**2.88 ± 1.05**	**1.52 ± 0.59**	**0.002**
[Ac] _end of exercise_ (mmol/L)	**3.48 ± 1.38**	**1.21 ± 0.51**	**≤ 0.001**
Δ [Ac] _recovery-exercise_ (mmol/L)	0.10 ± 1.02	2.23 ± 1.97	
IMCL (% of water peak)	**0.33 ± 0.20**	**0.58 ± 0.26**	**0.04**
^31^***P*** ***MRS***			
[PCr] _at rest_ (mmol/L)	34.52 ± 4.96	29.04 ± 7.87	
[Pi] _at rest_ (mmol/L)	2.63 ± 0.57	2.52 ± 0.51	
pH _rest_	7.04 ± 0.03	7.03 ± 0.03	
ADP _at rest_ (µmol/L)	8.72 ± 0.63	8.60 ± 0.92	
PDE (mmol/L)	**2.12 ± 0.68**	**2.75 ± 0.52**	**0.04**
GPC (mmol/L)	**2.02 ± 0.64**	**2.68 ± 0.48**	**0.02**
PME (mmol/L)	0.28 ± 0.07	0.26 ± 0.07	
NADP(mmol/L)	0.20 ± 0.08	0.31 ± 0.18	
τ_PCr on-kinetics_ (s)	**44.74 ± 4.10**	**53.71 ± 5.68**	**0.002**
τ_PCr recovery_ (s)	**38.84 ± 7.6**	**71.02 ± 15.00**	**≤ 0.001**
Q_max_ (mM/s)	**0.76 ± 0.18**	**0.46 ± 0.21**	**0.004**
PCr drop (%)	43.54 ± 9.68	35.44 ± 11.35	
[PCr] _end of exercise_ (mmol/L)	17.97 ± 2.62	18.14 ± 3.44	
[Pi] _end of exercise_ (mmol/L)	6.98 ± 2.66	5.76 ± 2.81	
pH _end of exercise_	6.97 ± 0.03	6.99 ± 0.04	
ADP _end of exercise_ (µmol/L)	49.70 ± 14.60	54.20 ± 22.84	

Significant values are in bold.

Data are given as mean ± standard deviation.

A between-group changes in acetylcarnitine metabolism over time was detected, therefore differences between groups depend on the time point. At rest, we observed significantly lower acetylcarnitine concentrations in the overweight/sedentary group than in the lean/active group (overweight/sedentary: 1.52 ± 0.59 mmol/L, lean/active: 2.88 ± 1.05 mmol/L; *p* = 0.003). There were significant differences between the overall averaged acetylcarnitine concentrations between the two groups (*p* < 0.001) at almost every interval during exercise and recovery, except for the last two intervals (15 and 23 min after exercise) (Fig. 2C). Further, in the lean/active group, acetylcarnitine content started to decay upon cessation of exercise, while, in the overweight/sedentary group, acetylcarnitine content continued to rise for the next 15 min, after which it reached a plateau until the end of the measurement time (Fig. 2C). The lean/active group reached the highest concentrations right after exercise (5.7 ± 1.63 mmol/L), whereas the overweight/sedentary group reached highest concentrations 15 min after exercise (3.49 ± 2.25 mmol/L).

In the dynamic ^31^P MRS data, as expected, PCr concentrations decreased during exercise, reached a steady state, and recovered toward basal values after exercise in all volunteers (Fig. 3A). The plantar flexion exercise protocol of 15 min at 30–50% of the individuals’ predetermined MVC yielded a PCr depletion of 40.5% ± 11.3% in all subjects.

The ADP metabolism differed over time between the groups (*p* = 0.029) (Fig. 3B). Prior to exercise, ADP levels (μM) at rest were similar across the groups. At the onset of exercise, ADP levels increased rapidly in both groups (Fig. 3B). When PCr reached a steady state during exercise, we observed a trend toward lower end-exercise ADP in lean/active subjects but not in overweight/sedentary subjects, which did not reach significance (p = 0.06).

No significant correlations between acetylcarnitine and PCr, Pi, and ADP concentrations were found.

**Figure 4:**
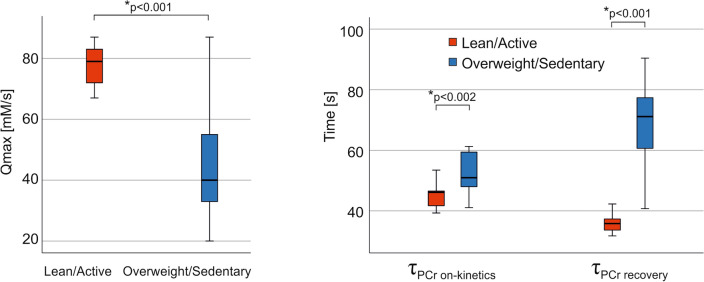
Boxplots showing the difference of Q_max_, τ_PCr on-kinetics,_ and τ_PCr recovery_ between lean and active (red) and overweight and sedentary (blue) volunteers.

In vivo skeletal muscle mitochondrial capacity, as determined by the rate constant of PCr resynthesis after exercise (τ_PCr recovery_), was significantly different across the groups (*p* < 0.001), with a significantly slower PCr recovery in the overweight/sedentary group compared to the lean/active group. Likewise, significant differences in τ_PCr on-kinetics_ (PCr at the onset of exercise) were observed between those two groups of volunteers (*p* = 0.002), with a slower τ_PCr on-kinetics,_ again, in the overweight/sedentary group. In addition, in this group, a significantly lower Q_max_ (*p* = 0.002) was observed (Fig. 4).

In addition to the results from the dynamic, interleaved protocol, we evaluated static ^1^H and ^31^P MR spectra and found significant differences in IMCL and PDE between the lean/active and the overweight/sedentary groups (both *p* = 0.04), with higher IMCL content and higher GPC concentrations in the overweight and sedentary group.

### Correlations between the measured parameters

**Figure 5:**
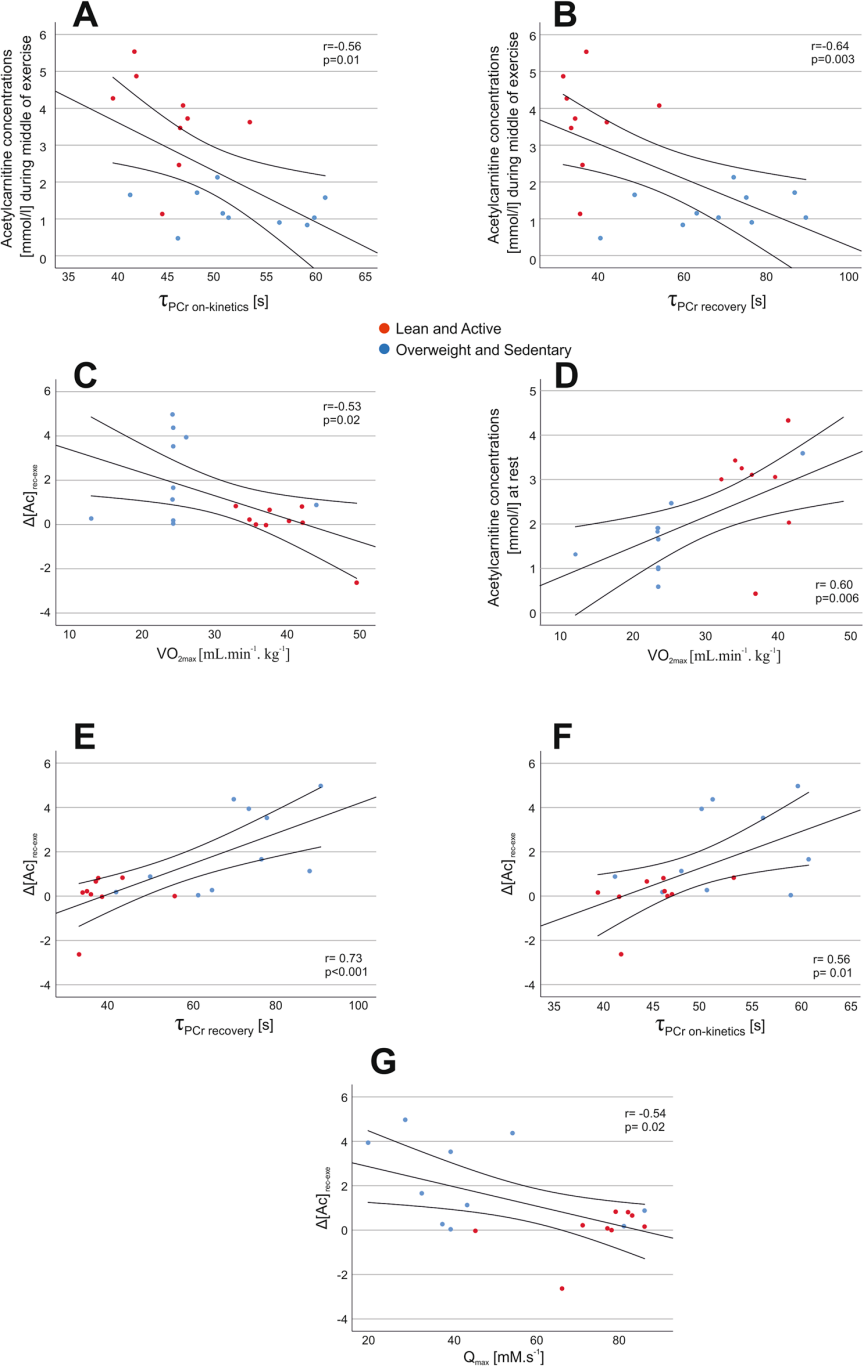
Linear relations between τ_PCr on-kinetics_, τ_PCr recovery,_ and in vivo skeletal muscle acetylcarnitine content during exercise (**A**, **B**), and between Δ[Ac] rec-exe and VO_2max_, τ_PCr on-kinetics_, τ_PCr recovery,_ and Q_max_ (**C**, **E**, **F**, **G**) and acetylcarntiine concentrations at rest and VO_2max_ (**D**).

Linear relations between τ_PCr on-kinetics_, τ_PCr recovery_, VO_2max,_ and in vivo skeletal muscle acetylcarnitine content are shown in Fig. 5A–G. Furthermore, GPC positively correlated with BMI (*r* = 0.46, *p* = 0.04) and negatively correlated with VO_2max_ and Q_max_ (*r* = *− *0.60,* p* = 0.006;* r* =  *− *0.58,* p* = 0.009), while IMCL and BMI were positively correlated (*r* = 0.56,* p* = 0.01) and IMCL positively correlated with both GPC and PDE (*r* = 0.53,* p* = 0.02;* r* = 0.50,* p* = 0.03). Another interesting correlation was between τ_PCr on-kinetics_ and τ_PCr recovery_, which were both negatively correlated with VO_2max_ and MVC (τ_PCr on-kinetics_ vs. VO_2max_: *r* = − 0.68, *p* = 0.001; τ_PCr recovery_ vs. VO_2max_: *r* =  − 0.74,* p* < 0.001; τ_PCr on-kinetics_ vs. MVC: *r* =  − 0.50,* p* = 0.02; τ_PCr recovery_ vs. MVC: *r* =  − 0.61,* p* = 0.005). Moreover, V_PCr_ positively correlated with both VO_2max_ and MVC (*r* = 0.62,* p* = 0.004;* r* = 0.67,* p* = 0.001).

## Discussion

Acetylcarnitine as a relatively low-concentration metabolite that fulfills a major role in maintaining pyruvate dehydrogenation activity. This is further connected to whole-body glucose homeostasis and metabolic flexibility^3^, and is, therefore, of high interest in skeletal muscle research. It is already known that a heavy exercise workout leads to increased acetylcarnitine concentrations immediately after the exercise challenge^17^; however, the in vivo behavior of muscle acetylcarnitine content during exercise with MRS has not been shown.

To the best of our knowledge, we are the first to show in vivo changes of skeletal muscle acetylcarnitine during submaximal acute exercise (aerobic exercise) and immediate recovery from exercise using a protocol that can be tolerated by a broad range of volunteer and patient populations. The straightforward detection and quantification of acetylcarnitine is challenging, due to the strong overlap of the 2.13-ppm line with lipid resonances, but the differences in T_2_ relaxation times of acetylcarnitine and lipids and superior spectral resolution at 7 T allow for the detection of the 2.13-ppm line at rest, using a long-TE ^1^H MRS^1,2^, even in overweight and sedentary volunteers (Fig. 2B). In some of overweight group the visibility of acetylcarnitine peak can be limited due to the spectral overlap with adjanced lipid resonances which in general are more pronounced. Another added value of this reasearch is that we have assessed skeletal muscle acetylcarnitine together with PCr during a single dynamic session using interleaved ^1^H/^31^P MRS. We were, therefore, able to quantify both time courses in one single experiment, which allowed for the measurement of different parameters under a unique exercise load and metabolic conditions (which would otherwise be difficult or impossible to reproduce in the exact same fashion)^25^. It has the further benefit of increasing volunteer compliance with the experimental procedures, due to a shortened protocol, compared to consecutive acquisitions. We examined metabolites from gastrocnemius medialis and lateralis muscles. One can argue that ^31^P DRESS slab as applied here includes small parts of soleus muscle as well, however plantar flexion with the straight leg is described as a suitable model for dynamic data collection from gastrocnemius muscles^40^. In other words, it is true that a small fraction of soleus tissue contributes to the total signal, but because soleus is mostly inactive, it only produces a (small) constant offset to the metabolite signals of PCr, Pi and ATP, with minor influence on recovery rates^41^.

A between-group difference in acetylcarnitine metabolism over time was detected. First, we observed significantly lower acetylcarnitine concentrations in the overweight/sedentary group than in the lean/active group. Lindeboom et al.^1^ found similar results in the vastus lateralis muscle with 3 T MRS, where obese, sedentary subjects had lower acetylcarnitine concentrations than lean subjects (lean sedentary subjects: 1.28 ± 0.22 mmol/kg_ww_; obese sedentary subjects: 0.70 ± 0.22 mmol/kg_ww_). In our research^16^ focused on the soleus and tibialis anterior muscles at 3 T, we found similarly lower acetylcarnitine content in impaired glucose tolerance (IGT) patients than in volunteers with normal glucose tolerance (NGT) (SOL: IGT: 1.9 ± 0.2 mmol/L tissue volume vs. NGT: 2.3 ± 0.6 mmol/L tissue volume, TA: 0.9 ± 0.4 mmol/L tissue volume vs. 1.2 ± 0.8 mmol/L tissue volume). The BMI of this IGT group was similar to the BMI of the overweight and sedentary group in the present study. It is difficult to compare the exact concentrations; however, calculated concentrations in mmol/L units are slightly higher than in mmol/kg_ww,_ as they were corrected for the specific weight of skeletal muscle tissue. Furthermore, here we investigated another type of muscle, i.e., the gastrocnemius medialis and lateralis, in which the proportion of type I and type II muscle fibers is typically 50:50^42,43^. This is more comparable to the vastus lateralis muscle than to the soleus muscle (predominantly type I fibers)^43^ and the m. tibialis anterior (predominantly type II fibers)^44^.

We report resting acetylcarnitine concentrations of 1.5 to 2.8 mmol/L, which is in good agreement with vast majority of results of biopsy studies^9–14,45^ when these are converted to mmol/L (ranging from ~ 0.5 to 4.8 mmol/kg dry mass). These studies reported acetylcarnitine concentrations assessed from biopsy samples after 10-, 30- and 60-min of high intensity exercise (70, 80 and 65% VO_2max_ resp.), where acetylcarnitine content increased up to 15 mmol/kg dry mass^12–14^. We are not able to compare acetylcarnitine concentrations from such a high intensity exercise challenge as the goal of our exercise challenge is to maintain aerobic conditions to avoid acidifying of muscles, peak-splitting of the Pi resonance in ^31^P MR spectrum during exercise and bi-exponential behaviour of PCr recovery.

When we looked at the time course of acetylcarnitine (2 min rest, 15 min exercise, 23 min recovery) in both groups, we recognized significant differences between the overall means of acetylcarnitine concentrations between those two groups at almost every interval during the exercise and recovery phases, except for the last two points (15 min of recovery and end of recovery) (Fig. 2C), which may indicate distinct states of skeletal muscle metabolism. It is known that obesity and T2DM affect the regulation of muscle fat oxidation, particularly during exercise, and carnitine availability may limit fat oxidation^46^. The uptake and/or oxidation of fatty acids have also been shown to be impaired during post-absorptive conditions in overweight subjects and/or in subjects with T2DM. Also, human studies have shown that the muscle of subjects who are overweight and/or have T2DM is characterized by an inability to increase fatty acid uptake and/or fatty acid oxidation during exercise^47–50^.

Regarding the recovery period for acetylcarnitine content, in the lean/active group, the increased level of acetylcarnitine content after exercise started to decay, suggesting increased consumption, while, in the sedentary/overweight group, levels of acetylcarnitine continued to rise (Fig. 2C). Similar observations were found by Seiler et al.^33^ in which muscle levels of acetylcarnitine were measured in trained and untrained subjects (n = 4) by ^1^H-MRS before, 15 min after, and during recovery from 30 min of cycle ergometer exercise performed at 50% of maximal workload.

Further, in our volunteers, the lean/active group reached the highest acetylcarnitine concentrations shortly after exercise, whereas, in the overweight/sedentary group, the acetylcarnitine content increased during a longer period, reaching its maximum 15 min after exercise. Previous studies have shown that acute exercise can lead to an increase in acetylcarnitine concentrations in skeletal muscle^51,52^. The surge is believed to be a reaction to the increased requirement for energy generation from fatty acids while exercising^52,53^. Variations in the timing of the highest acetylcarnitine levels during recovery and the degree of wash-out could indicate the intensity and length of prior exercise, along with physiological distinctions among the observed muscle groups. Time series of unaveraged acetylcarnitine evolution during exercise/recovery period from all volunteers is shown in supplementary figure. 

In skeletal muscle, non-invasive ^31^P MRS measurements of the post-exercise recovery kinetics contain valuable information about muscle mitochondrial function and cellular pH homeostasis in vivo^24,54^. The method has been an established research tool with which to assess muscle energy metabolism and to distinguish between different metabolic and pathologic states. In our dynamic ^31^P MRS data, we observed decreased PCr concentrations during exercise which reached a steady state and recovered toward steady state values after exercise. Fifteen minutes of plantar flexion exercise yielded a PCr depletion of 40.5% ± 11.3% in all subjects. MVC was used as a measure to set the exercise workload, as this takes into account direct power production of the muscle involved in the given exercise. Yet, one could contend that we overlooked the variation in power output associated with the lactate threshold. However, the measured intracellular pH at the end of exercise did not show substantial acidosis in either of the groups (pH_end of exercise_ in overweight/sedentary: 6.99 ± 0.04; lean/active: 6.97 ± 0.03). Individuals who are trained exhibit increased maximal aerobic power, as evidenced by their elevated VO_2max_ scores. This signifies their enhanced ability to transport oxygen to muscles and facilitate its transfer to mitochondria, where it acts as an electron acceptor during ATP production. In a review by McMahon and Jenkins, the authors suggest that people boasting a higher VO_2max_ are expected to have improved capacity in replenishing PCr after engaging in intense exercise^55^. The linear relations we found between τ_PCr_ (both at on-kinetics and recovery), V_PCr,_ and VO_2max,_ as well as with MVC, appear to corroborate these results consistently, with the notion that elevated VO_2max_ results in a faster PCr resynthesis. As a result, it is not surprising that the rate of PCr resynthesis following moderate exercise represents the muscle’s oxidative capacity, which has been found to be faster in endurance-trained athletes, compared to sedentary controls^55^. Our results from dynamic ^31^P MRS are consistent with this statement, since τ_PCr_ (both at on-kinetics and recovery), was significantly slower in the overweight/sedentary group compared to the lean/active group. In addition, in the overweight/sedentary group, there was a significantly lower Q_max_.

The further value of this interleaved ^1^H/^31^P MRS protocol is shown in the ability to calculate the time course of ADP concentrations^39^. Earlier findings indicated a decrease in basal ADP levels and reduced sensitivity to ADP in individuals facing metabolic challenges or those in older age groups^56,57^. Likewise, Mancilla et al. reported higher ADP levels during exercise in T2DM patients and obese patients compared to lean/trained individuals^21^. Our observations were very similar, since we found that ADP metabolism differed over time between the groups. While pre-exercise ADP levels were comparable across the groups, we observed a marginal difference in ADP levels across the groups in the time point when PCr reached a steady state during exercise. Although the difference at this time point was not significant, the difference in the whole-time course of ADP metabolism was found to be significant between the groups. Mancilla et al. proposed that increased ADP levels are necessary to activate the mitochondrial oxidative phosphorylation system in groups facing metabolic challenges. This could potentially offer an alternative mechanistic rationale for the disparities observed in PCr kinetics among groups at the start of exercise^21^. These findings could imply that metabolically compromised individuals require greater metabolic stress to trigger oxidative ATP production, leading to an extended reliance on substrate-level phosphorylation^21^. Moreover, in their work, their findings demonstrated a strong correlation between CrAT protein activity in muscle tissue and PCr kinetics at the start of exercise. Additionally, metabolically compromised individuals exhibited reduced skeletal muscle acetylcarnitine content at rest, which significantly correlated with slower PCr kinetics when exercise began^21^.

In this work, we found linear relations between τ_PCr_ (both at on-kinetics and recovery) and in vivo skeletal muscle acetylcarnitine content during exercise. Furthermore, Δ[Ac] _rec-exe_ was positively correlated with τ_PCr_ (both at on-kinetics and recovery) and with Q_max_. These discoveries may indicate that the acetylcarnitine content within skeletal muscle influences mitochondrial inertia and acts as a trigger for PCr kinetics during exercise.

It has already been shown, and here we re-confirm, that acetylcarnitine content and CrAT protein activity were lower in T2DM and obese individuals. This supports the notion that reduced CrAT protein activity-and therefore, a low capacity to form acetylcarnitine from Acetyl-CoA and carnitine-might underlie metabolic inflexibility and impaired insulin sensitivity^1^. Importantly, the CrAT enzyme also functions in the reverse direction to supply Acetyl-CoA from acetylcarnitine when energy demand suddenly increases.

In addition, we found relation between basal acetylcarnitine levels and VO_2max_. This connection may be based on the facts that rate of oxidative phosphorylation from human skeletal muscle is in very close correlation with VO_2max_^58,59^. Myoglobin transports oxygen to the mitochondria where it can be used as the last electron acceptor within oxidative phosphorylation, allowing for ATP synthesis to occur. During transitions from low to high exercise workloads, Krebs cycle flux must increase to keep pace with the high ATP demands of muscle contraction. Here CrAT functions to sustain high rates of oxidative ATP regeneration by acting as a channel for acetyl group transfer^60^. Elevated levels of acetylcarnitine might therefore indicate better mitochondrial efficiency, leading to improved energy production during aerobic activities, which could be in good agreement with better aerobic fitness.

Limitaition is, that all correlations are shown from both groups (lean/active and overweight/sedentary) and analysis of individual groups may be important. However, this correlation anaylsis detected trends but did not reach significancy through variables probably owing to the point that individual groups contained 10 and 9 subjects only.

Nevertheless, from our results, we cannot draw general conclusions about exact changes and the exercise effects on skeletal acetylcarnitine content in the insulin-resistant and diabetic population. This is because our study included only a limited number of volunteers and was aimed mainly at the methodological aspects to allow testing the dynamic data acquisition and exploring the behavior of in vivo skeletal muscle acetylcarnitine, as well as the relation to phosphocreatine during submaximal plantar flexion exercise using interleaved ^1^H/^31^P MRS at 7 T. Further studies on the influence of food intake, diet composition, sex, age, and different pathophysiological conditions with a larger number of volunteers with a broader range of metabolic conditions and physical fitness are necessary for detailed analysis.

In conclusion, we are the first to show in vivo changes of skeletal muscle acetylcarnitine during acute exercise and immediate exercise recovery with a submaximal aerobic workload using interleaved ^1^H/^31^P MRS at 7 T that can be tolerated by a broad range of volunteer and patient populations. Moreover, we report on between-group differences in changes of acetylcarnitine metabolism over the rest/exercise/recovery periods, as well as significantly lower Q_max_ and slower τ_PCr_ (both at on-kinetics and recovery) in an overweight/sedentary group compared to a lean/active group. Furthermore, we report cross-linked relationships between τ_PCr on-kinetics_, τ_PCr recovery_, Q_max_, VO_2max_ and in vivo skeletal muscle acetylcarnitine.

### Supplementary Information

Supplementary Information.

Supplementary Figures.

## Data Availability

Anonymized MR (Magnetic Resonance) data acquired at MR scanner are copied from the scanner and are stored at the secured internal servers provided by the beneficiary with access restricted to the researchers involved in the study. All persons who receive access to encrypted and non-encrypted data are subject to the data protection basic regulation (DSGVO) as well as the Austrian adaptation regulations in the respectively valid version. Those datasets are available from the corresponding author on reasonable request. Non-encrypted data will not be available to protect study participant privacy.

## Abbreviations

CrAT: Carnitine acetyltransferase
CK: Creatine kinase
TE: Echo time
GM: Gastrocnemius muscle
GPC: Glycerolphoshocholine
GPE: Glycerolphosphoethanolamine
IGT: Impaired glucose tolerance
Pi: Inorganic phosphate
VO_2max_: Maximal oxygen uptake
MVC: Maximal voluntary contraction force
Q_max_: Maximum oxidative capacity
NADP: Nicotinamide adenine dinucleotide phosphate
NGT: Normal glucose tolerance
NA: Number of averages
PE: Phopshoethanolamine
PCr: Phosphocreatine
PDEs: Phosphodiesters
PMEs: Phosphomonoesters
PC: Phosphocholine
^31^P MRS: Phosphorus magnetic resonance spectroscopy
^1^H MRS: Proton magnetic resonance spectroscopy
TR: Repetition time
τ_PCr__on__-kinetics_: The time constant of PCr decay at the onset of exercise
τ_PCr__recovery_: The time constant of PCr resynthesis
T2DM: Type 2 diabetes mellitus
VOI: Volume-of-interest
