# Supplementary material for: Correlation between skeletal muscle acetylcarnitine and phosphocreatine metabolism during submaximal exercise and recovery: interleaved 1H/31P MRS 7 T study
Source: Sci Rep. 2024 Feb 8;14:3254. doi: 10.1038/s41598-024-53221-x (PMC10853526; doi:10.1038/s41598-024-53221-x)
Supplement: Supplementary file 1 — Supplementary Information. [file 41598_2024_53221_MOESM1_ESM.docx]

MRS checklist

| 1. Hardware |  |
| --- | --- |
| a. Field strength [T] | *7 T* |
| b. Manufacturer | *Siemens Healthineers, Erlangen, Germany* |
| c. Model (software version if available) | *Magnetom Tera dot Plus 7T (VE12U)* |
| d. RF coils: nuclei (transmit/receive), number of channels, type, body part | *Custom-built three channel ^31^P (d = 15 cm, l = 10 cm), two channel ^1^H (d = 17 cm, l = 12.5 cm) transceiver coil, shaped to the human calf, (Ref: Goluch et al. Magn Reson Med 2015;73(6):1190–1195.)* |
| e. Additional hardware | *Trispect ergometer (Ergospect, Innsbruck, Austria) dedicated for plantar flexion exercise* |
| 2. Acquisition |  |
| a. Pulse sequence | *Temporally interleaved Semi-LASER for ^1^H MRS with slab-localized (DRESS) for ^31^P MRS* |
| b. Volume of interest and VOI locations | *^1^H VOI-Single voxel placed obliquely in gastrocnemius medialis and lateralis muscle, avoiding subcutaneous fat, fasciae and adjacent muscles*  *^31^P VOI- slab placed covering g. medialis and g. lateralis* |
| c. Nominal VOI size [cm^3^, mm^3^] | *^1^H MRS: Anatomy-matched, 13x20x40 mm^3^*  *^31^P MRS: slab thickness of 18 mm* |
| d. Repetition Time (TR), Echo Time (TE) [ms, s] | *^1^H MRS: TR = 6 s, TE = 300 ms*  *^31^P MRS: TR = 6 s, acquisition delay 2.4 ms* |
| e. Total number of excitations or acquisitions per spectrum (NA)  In time series for kinetic studies   1. Number of averaged spectra) per time-point (NA) 2. Averaging method (e.g. block-wise or moving average)   Total number of spectra (acquired / in time-series) | *^1^H: NA= 20*  *^31^P rest: NA= 16*  *Total number of spectra in time series was 400* |
| f. Additional sequence parameters (spectral width in Hz, number of spectral points, frequency offsets)   1. If STEAM:, Mixing Time (TM) 2. If MRSI: 2D or 3D, FOV in all directions, matrix size, acceleration factors, sampling method | *^1^H: 5 kHz, 2048 complex points after removing oversampling*  *^31^P: 5 kHz, 2048 complex points after removing oversampling* |
| g. Water suppression method | *^1^H: WET water suppression* |
| h. Shimming method, reference peak, and thresholds for “acceptance of shim” chosen | *1H: 3D shim & individual Interactive shim, linewidth <45Hz* |
| i. Triggering or motion correction method  (respiratory, peripheral, cardiac triggering, incl. device used and delays) | *Volunteers were instructed to press the pedal twice between measurements, with noise of the spoiler gradients serving as audio cue, to ensure data acquisition in a relaxed state of the muscle* |
| 3. Data analysis methods and outputs |  |
| a. Analysis software | *All ^1^H/^31^P spectroscopy data were extracted and processed from raw data using in-house developed Python scripts (http://www.python.org) and for phasing and channel combination.*  *Signals were phased to the highest peak magnitude of PCr/ water in the frequency domain after 7 Hz Lorentzian apodization and 4 × zero-filling. The channel combination was then performed by weighted averaging of the raw data (that is, without apodization and zero-filling).*  *All spectra were analyzed with the fitting routine AMARES, using jMRUI v6.0 alpha* |
| b. Processing steps deviating from quoted reference or product analysis software (vendor, version) | *Gaussian line shapes, soft constraints for frequencies* |
| c. Output measure  (e.g. absolute concentration, institutional units, ratio) Processing steps deviating from quoted reference or product | *Concentrations in institutional units and pH values*  *Acetylcarnitine in mmol/L tissue volume*  *IMCL as percentage of water content*  *^31^P metabolites mmol/l* |
| d. Quantification references and assumptions, fitting model assumptions | *^31^P MR: Quantification relative to ATP, which was assumed to be constant ( [ATP] equal to 8.2mM )*  *depletion during the exercise and end-exercise PCr depletion relative to post-exercise asymptotic value of mono-exponential fit of recovery*  *^1^H MR: Quantification relative to water, fully relaxed water signal was measured separately using the same sequence parameters without water suppression* |
| 5. Data Quality |  |
| a. Reported variables  (SNR, Linewidth (with reference peaks)) | *SNR taken from AMARES results, noise from residue*  *Linewidths of acetylcarnitine were taken from the AMARES results.* |
| b. Data exclusion criteria | *Not visible acetylcarnitine peak in ^1^H MR spectra* |
| c. Quality measures of postprocessing Model fitting (e.g. CRLB, goodness of fit, SD of residual) | *SD of residual* |
| d. Sample Spectrum | *Figure 2A, 2B* |
